# A methodology for calculating the rarity of diverse proteins based on functional specificity and thermodynamic stability

**DOI:** 10.1371/journal.pone.0339572

**Published:** 2025-12-29

**Authors:** Brian J. Miller

**Affiliations:** Biologic Institute, Redmond, Washington, United States of America; UNC CH: The University of North Carolina at Chapel Hill, UNITED STATES OF AMERICA

## Abstract

A key question in protein studies is the proportion of amino acid sequences that correspond to functional proteins, often called protein rarity. This issue underlies the relationship between mutations and disease, theories on the origin of proteins, and strategies for engineering new proteins. Recent literature has detailed how to employ estimates of protein rarity to evaluate the required biasing of functional sequences in sequence space to allow for evolutionary paths to connect distinct proteins. One challenge in addressing rarity has been an imprecise definition of function and a lack of consistency in methodology. This study introduces a new methodology, referred to as PRISM, to evaluate protein rarity based on the impact of mutations on stability. PRISM offers a suite of methods that are simpler than traditional approaches while providing accurate upper-bound rarity estimates. The specific method applied is determined by the protein’s function and available empirical data on how accumulating mutations affect its stability and performance. PRISM is applied to several proteins, and the accuracy of the methods is demonstrated by comparing the results to rarity estimates from previous studies. The calculated rarities align with previous research that concludes functional sequences are often exceedingly rare. The application of PRISM is outlined for research in protein engineering, protein evolution, and pathology.

## Introduction

### Protein rarity studies

A fundamental question in protein studies is the rarity of amino acid (aa) sequences in sequence space (aka “protein rarity”) that generate functional protein folds. Sequence space is the high-dimensional space of all possible amino acid sequences or nucleotide sequences in the corresponding open reading frame (ORF) in DNA, and a fold is a protein’s three-dimensional structure that allows it to perform its function or functions. Protein rarity can be defined as the proportion or prevalence of functional sequences, $P_{fs}$, in a region of sequence space, which equals the number of amino acid sequences that fold into the correct structure to perform a specific function divided by the total number of sequences within that region. Global rarity corresponds to the region encompassing all of sequence space.

Rarity calculations constrain theories on the origin of natural proteins and strategies for engineering new proteins [1,2]. For instance, they yield estimates on the required biasing in sequence space for continuous paths of functional sequences to connect distinct proteins [3]. In addition, identifying the underlying constraints for protein function and their relationship to rarity assists in developing antibiotics [4] and enables better predictions of why genetic variants cause disease [5].

One class of experiments addressing protein rarity uses a forward approach where random sequences are selected for some predefined function, such as the ability to bind to ATP [6–10]. Such experiments frequently report that functional sequences are relatively common. However, the identified amino acid chains in these experiments only perform very simple activities. They lack the suite of properties often associated with natural proteins, such as the precise positioning of side chains to perform targeted molecular alterations and cooperative conformational changes that allow for complex catalytic and mechanical functions [11].

Other experiments use a reverse approach, where wildtype proteins are tested for how much randomization they can withstand and remain viable [12–15]. These studies consistently conclude that functional sequences corresponding to natural proteins are extremely rare. Yet experimental approaches are, by necessity, limited by the amount of sequence space investigators can sample, which is a minuscule portion.

Sequences are mostly sampled in the region of sequence space close to a wildtype protein sequence, so estimates do not include substantive sampling of sequences with a larger number of amino acid alterations. The number of alterations, n, is known as the Hamming distance from the wildtype. Since sequences close to wildtype sequences (i.e., small n) are more tolerant to amino acid changes, researchers risk overestimating functional prevalence [1,16]. In addition, the extent to which results can be applied more generally to other proteins is uncertain. A more reliable approach for estimating rarity focuses on changes in protein stability and functional efficiency caused by accumulating mutations.

This study has three primary aims: (1) to develop a generalizable framework—PRISM (Protein Rarity Inference from Stability and Mutational data)—for estimating protein rarity based on mutational effects on function and thermodynamic stability; (2) to apply PRISM to a diverse set of well-characterized proteins representing a range of structural and functional types; and (3) to evaluate the framework’s performance by comparing its rarity estimates with those derived from previous empirical and computational studies.

### Protein stability studies

**Fig 1:**
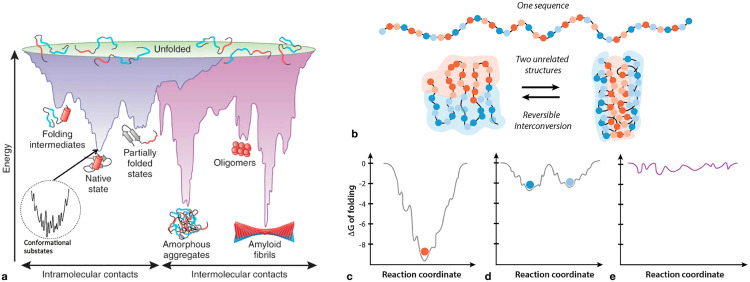
Energy landscapes for different protein categories. **(a)** A protein’s energy landscape illustrating the unfolded, native, intermediate, and alternative states. The native state represents the free energy minimum for proteins under standard conditions. Protein folding corresponds to an unfolded amino acid chain (depicted at the top of the diagram) “funneling” down the energy landscape into a minimum energy basin via intramolecular contact formation. The basin contains numerous conformational substates. Local energy minima represent intermediate or alternative conformations, such as the amyloid state, which can occur if the correct environmental conditions are present. Illustration reproduced from Rashkatov and Teplow (2017) [19], licensed under CC BY 4.0. **(b)** Metamorphic protein transitioning between two distinct conformations. **(c)** Protein with a single native state corresponding to a global energy minimum. **(d)** Metamorphic protein with two conformations corresponding to separate energy minima. **(e)** Intrinsically disordered protein lacking a well-defined structure. Subfigures (b–e) adapted from Dishman and Volkman (2022) [20], licensed under CC BY 4.0.

Protein stability has been traditionally defined as the free energy difference between the unfolded and native state: ${\Delta G}_{U-N}$. For instance, β-lactamase has a stability of approximately 11 kcal/mol [17]. Sorokina et al. (2022), in contrast, define protein stability as the depth of a local free energy minimum [18]. The preferred definition does not affect the analysis presented here. An unfolded chain “funnels” down the protein’s energy landscape (Fig 1a) toward the energy minimum corresponding to the native conformation, whose depth is ${\Delta G}_{U-N}$.

Zeldovich et al. (2007) reported that most proteins have a stability of 15 kcal/mol or less, with the highest recorded stabilities around 20 kcal/mol [21]. The investigators derived this range using data obtained from the ProTherm database, which contains free-energy changes (ΔΔG) resulting from mutations in proteins from diverse species. The database also includes ${\Delta G}_{U-N}$ for several thousand proteins. The investigators fit the ΔΔG data to mutation-diffusion equations to derive the distribution of protein stabilities. They also directly constructed the protein stability distribution from the $\;{\Delta G}_{U-N}$ recorded in the database. The derived distribution matches the distribution from the empirical data, demonstrating the accuracy of the stability range they reported.

A key result from multiple studies is that the extent to which a mutation lowers a protein’s stability is very closely associated with the extent to which it degrades performance [22–24]. When the stability drops sufficiently, activity ceases. The average drop in stability due to a mutation (ΔΔG) is approximately 1 kcal/mol [25].

A second key result is that accumulating mutations often exhibit epistasis [1,26,27], which can be categorized into general and specific [28]. General epistasis refers to mutations that alter the physical properties of proteins (e.g., affinity for ligands, stability) in an additive manner but impact the performance non-additively due to the nonlinear relationship between the physical properties and their biological effects. Specific epistasis refers to mutations that physically interact such that their impact on physical properties is nonadditive. Positive specific epistasis can occur if one mutation undoes the negative impact of another, but negative specific epistasis is far more prevalent [28].

The most important implication of negative specific epistasis for PRISM methods is that fewer mutations are tolerated as mutations accumulate, so the local *P*_*fs*_ decreases rapidly with *n*. As mutations accumulate, they increase the probability that the next mutation will interact epistatically with an existing mutation, thus exacerbating the drop in stability. The combined effect of general and specific epistasis is that a drop in stability does not initially decrease performance significantly, but after a threshold is exceeded, performance quickly degrades with accumulating mutations [1]. As a result, nearly all functional sequences reside within a target region in sequence space bounded by a specific Hamming distance from the wildtype, $n_{b}$.

### Methodology for estimating rarity

PRISM incorporates a spectrum of novel methods for calculating protein rarity from the available data on the loss of stability and function caused by accumulating mutations. The schema maps to a process flowchart that uses a decision tree to determine the specific method. The choice of method is partially determined by the protein’s functional specificity, which represents the precision required in the location and orientation of a protein’s amino acids to perform a biological function at a level that benefits the organism. The available empirical data also determines the selected method and the values of the corresponding parameters. Since most of the methods are based on the physics of protein stability, they generate more reliable estimates that are not limited by sampling constraints.

The first branch in the flowchart separates peptides, polypeptides, and simple proteins that perform low-specificity functions from proteins that perform high-specificity functions. Examples of low-specificity functions include simple binding, breaking weak chemical bonds, and catalyzing reactions that simple molecules can catalyze. For low-specificity proteins, $P_{fs}$ can often be directly measured. High-specificity functions include breaking strong bonds, multistep reactions, and driving processes that require the protein to perform multiple highly coordinated conformational changes or precise interactions with other proteins. For high-specificity proteins, $P_{fs}$ cannot be measured directly.

**Fig 2:**
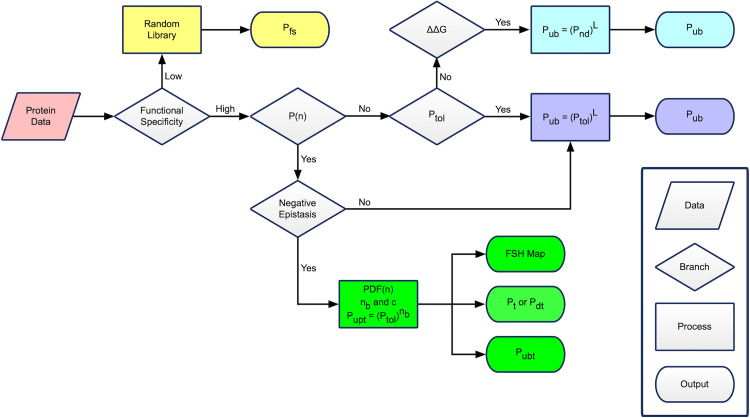
PRISM Flowchart for estimating protein rarity. The flowchart illustrates the decision tree used to determine methods for calculating rarity. The methods include (1) using a random library of amino acid sequences to directly estimate the percentage of functional sequences, $P_{fs}$ (yellow); (2) using the proportion of functional sequences with n altered amino acids, P(n)*,* to (2a) identify a target region and calculate the upper bound to the proportion of functional sequences in the target, $P_{ubt}$, & the probability of a random mutation residing in the target region, $P_{t}$*,* or (2b) calculate $P_{ubt}$ & the probability an undirected search could discover the target, $P_{dt}$, (green); (3) using the percentage of tolerated mutations, $P_{tol}$, to calculate the upper bound to the proportion of functional sequences, $P_{ub}$; and (4) using the change in protein stability due to mutations (ΔΔG) data to calculate $P_{ub}$ (purple). In the last method, the percentage of non-destabilizing mutations, $P_{nd}$, is used as a proxy for $P_{tol}$. Non-destabilizing mutations are categorized as those that decrease ${\Delta G}_{U-N}$ less than a threshold (ΔΔG <$G_{th}$).

The second branch separates high-specificity proteins based on whether P(n) can be defined—the probability that a protein is functional with n amino acid changes; if P(3) = 0.1, 10% of proteins with 3 altered amino acids from the wildtype are functional. The third branch separates proteins with known P(n) based on whether they display prevalent negative epistasis (aka epistatic proteins), and the fourth branch separates high-specificity proteins with unknown P(n) based on whether the percentage of tolerated mutations, $P_{tol}$, beyond a stability cutoff is known. Rarity is calculated for each category of proteins using the corresponding method specific to the demarcation criteria. The process flowchart is illustrated in Fig 2.

### Application of methodology

PRISM was employed for a wide variety of proteins. The method designated for epistatic proteins was applied to β-lactamase, green fluorescent protein (GFP), and HisA. These proteins provide the most comprehensive datasets in which the percentage of functional sequences was quantified across the greatest number of mutational steps without applying selection between rounds. In each experiment, large samples of clones were sequenced to identify the specific mutations within their protein-coding regions and to determine whether the resulting proteins retained function. These datasets therefore serve as ideal examples for demonstrating the method.

The method using ΔΔG distributions was implemented on the datasets for the 16 natural proteins analyzed by Tokuriki et al. (2007) [29] and 38 proteins in the FireProtDB database, a central repository of reported ΔΔG resulting from mutations [30]. The rarity measures generated by the different methods were compared to those reported by other studies to demonstrate PRISM’s broad applicability and general reliability. The discussion section examines general trends in protein rarity and explains how the methodology can be applied to future research.

## Methods

### Proteins displaying epistasis

The method designated for peptides, polypeptides, and low-specificity proteins entails generating a random library of amino acid sequences and directly measuring the proportion demonstrating functional activity. In contrast, the methods designated for high-specificity proteins rely on statistical analyses. The most accurate approach is applied when P(n) is known and demonstrates prevalent negative epistasis, fitting best to a hyper-exponential decay function:


$$P(n)=e^{-\alpha n-\beta n^{\text{2}}},$$ (1)

where *α* represents the initial exponential decline, and *β* represents the rate at which the exponential decay increases, thus the level of negative epistasis. Some of the most important data for this method is the level of negative epistasis, which directly determines β. The higher the value of β, the more quickly P(n) drops with increasing n (section A in S1 Text).

For epistatic proteins, nearly all the functional sequences reside within a target region in sequence space whose size is constrained by the β parameter. In practice, the target region or “target” refers to the neighborhood surrounding a wildtype sequence where upper bound estimates of $P_{fs}$ exceed the minimum probability threshold for discoverability, $P_{th}$. Outside the target, functional sequences are too rare for an unbiased search to ever find one, so $P_{fs}$ is effectively zero.

The threshold approximates the reciprocal of the maximum number of possible trials $T_{m}$, which is the total number of distinct gene variants that could have existed over Earth’s history. Assuming all organisms that have ever lived, $T_{m}$ approximates 10^38^, corresponding to $P_{th}\approx$ 10^-40^. For many multicellular eukaryotes, such as vertebrates, the effective threshold is closer to 10^-20^, reflecting their much smaller population sizes and longer generation times (see section B in S1 Text).

The target size is designated by the maximum percentage of nucleotides, c, or amino acids, $c_{a}$, that can differ from a reference sequence (e.g., wildtype protein sequence) for a sequence to reside within the target. The targets of most interest are c = 1/3 and c = 1/2, which correspond in amino acid sequence space to $c_{a}$ = 57 ± 3% and $c_{a}$ = 77 ± 3%, respectively (section B in S1 Text). This choice of targets aligns with empirical data and enables the direct application of Chatterjee et al. (2014) results to calculate target discovery times [31].

If, for a given protein, P(n) is known and exhibits prevalent negative epistasis, the designated method involves three sequential calculation steps to derive statistics describing protein rarity:

1. **Probability distribution of functional sequences**

The probability distribution function for functional sequences is derived and graphed to provide a qualitative depiction of how functional sequences are distributed across the Hamming distance from the wildtype.

2. **Estimation of the target boundary and search probabilities**

An upper bound for the target’s boundary is estimated. From this, two related quantities are determined:

a. The probability that a randomly selected sequence resides within the target region, and
b. The expected time required for a random search to discover the target. This time and $T_{m}$ directly yield the probability of a random search successfully discovering the target.

3. **Composite rarity estimation**

The probability that a randomly chosen sequence within the target is functional is then determined. Multiplying this probability by the target probability (from calculation 2a) yields a composite estimate of the protein’s rarity.

For the first calculation step, P(n) is used to generate PDF(n), which is the probability of a randomly chosen functional sequence differing from the wildtype sequence by *n* alterations:


$$PDF(n)=\frac{P(n)\cdot N_{seq}(n)}{N_{FS}},$$ (2)

where $N_{seq}(n)$ is the number of sequences differing from the wildtype by n alterations, and $N_{FS}$ is the total number of functional sequences. If the integral of PDF(n) from 10 to 60 is 0.2, then 20% of sequences that perform the wildtype function possess between 10 and 60 amino acid alterations (section A in S1 Text).

This study employs a new tool to graph PDF(n) called a *functional sequence heat* (FSH) map. It displays where in sequence space most functional sequences reside (Fig 2d). The FSH map portrays sequence space as a radial graph where the origin is the wildtype sequence, and the radial distance from the origin represents n. Larger values of PDF(n) are represented as darker colors since larger PDF(n) corresponds to a higher density of functional sequences with respect to n. The target region corresponds to the area of the map that encompasses all colored regions.

The FSH map depends on P(n), which is generated by curve-fitting experimental data up to a value of n much less than the protein’s length L. It is inaccurate for larger n since β changes with increasing *n* in a manner that has never been determined for any protein. Nevertheless, PDF(n) accurately depicts the general features of the distribution if negative epistasis is prevalent, so FSH maps for different proteins should provide a qualitative picture of their relative rarities. For proteins with similar lengths, the closer the dark regions are to the center, the greater the rarity.

For the second calculation step, the upper bound of the protein’s target, $n_{b}$, is determined from experimental data on the probability that a mutation will be tolerated, $P_{tol}$, after a protein’s stability drops beyond its stability threshold (section B in S1 Text):


$$n_{b}=\frac{\text{log}(P_{min}/P(n_{\text{0}}))}{\text{log}(P_{tol})}+n_{\text{0}},$$ (3)

where $n_{\text{0}}$ is the value of n where $P_{tol}$ was measured or calculated from P(n). An accurate measurement of $P_{tol}$ is critical for properly estimating $n_{b}$. Ideally, investigators should measure $P_{tol}$ by conducting mutational drift experiments both with and without selection between rounds. Those with selection should continue rounds until $P_{tol}$ plateaus; those without selection should continue until β can be reliably determined.

If investigators cannot experimentally determine $P_{tol}$ and P(n), the choice of $n_{\text{0}}$ will depend on the available data. Measurements of $P_{tol}$ for larger n will yield more reliable results, since proteins with few mutations are more tolerant of new mutations than proteins mutated past the stability threshold. If the mathematical model for P(n) closely matches the empirical data up to some n, that n can be used as $n_{\text{0}}$, and $P_{tol}$ can be set to $P(n_{\text{0}}+\text{1})/P(n_{\text{0}})$. Eq. 3 will overestimate the target boundary since this ratio typically decreases with n for proteins that display negative epistasis.

Another approach is to use a protein with a well-defined $P_{tol}$ as a benchmark. β-lactamase is particularly suitable since it performs a relatively simple catalytic reaction, and its $P_{tol}$ has been experimentally determined. Proteins that require greater precision to execute their function (i.e., perform more elaborate and difficult tasks) operate within tighter constraints, so they can be more easily disabled by mutations. If the protein under investigation performs a more challenging task than β-lactamase, β-lactamase’s $P_{tol}$ can be used in calculations. This choice can also be justified if the investigated protein displays greater negative epistasis (i.e., higher β) than β-lactamase.

If $n_{b}$ falls below the c = 1/3 boundary, the 1/3 boundary can be used in calculations since it yields a conservative upper bound to $P_{fs}$. This boundary also aligns with empirical studies of the minimum sequence identity (SI), where two proteins are likely to share the same general function—sharing the first three digits of an enzyme commission (EC) number [32]. In protein comparisons using FSH maps, the c = 1/3 boundaries can be marked on the maps as a visual cue to the relative protein sizes.

If $n_{b}$ falls above the c = 1/3 boundary but below the c = 1/2 boundary, the c = 1/2 boundary can be chosen since it matches empirical studies of the minimum sequence identity where two proteins share a similar structure [33,34]. It yields the most conservative rarity estimate. When β and $P_{\text{tol}}$ are determined with very high confidence, $n_{b}$ may be used as the target boundary. The most accurate estimate might come from P(n) or the FSH map, but it would not represent an upper bound.

The probability can be calculated for a randomly chosen sequence to reside within the target, $P_{t}$, by dividing the number of sequences in the target by the total number of sequences with the protein’s length:


$$P_{t}=\frac{\sum_{n=\text{0}}^{n_{b}}(\begin{array}{c} L\\ n \end{array}){\text{19}}^{n}}{{\text{2}\text{0}}^{L}}$$ (4)

Alternatively, the average discovery time, d, in number of trials can be calculated for a random search starting outside the target to find the target. Chatterjee et al. (2014) demonstrated that d follows an exponential function of L:


$$d=AB^{L}.$$ (5)

Here, the values for A and B can be determined by fitting the Chatterjee et al. (2014) data to Eq. 5. For c = 1/3, A = 2.21 and B = 3.25; for c = 1/2, A = 5.39 and B = 1.56 (section B in S1 Text):


$$d_{\text{1}/\text{3}}=\text{2}.\text{2}\text{1}\cdot (\text{3}.\text{2}\text{5}{)}^{L},$$ (6)

$$d_{\text{1}/\text{2}}=\text{5}.\text{3}\text{6}{\cdot (\text{1}.\text{5}\text{6})}^{L}.$$ (7)

The upper bound to the probability of a search discovering the target immediately follows:


$$P_{dt}=\text{1}-e^{-\frac{T_{m}}{d}}.$$ (8)

If a global view of rarity is desired, the best option is to calculate the probability of a sequence residing inside the target. Such an estimate could provide insight into the difficulty of engineering a protein that serves a similar purpose as a natural protein. Calculating the discovery time can aid in evaluating evolutionary scenarios.

For the third calculation step, the upper bound to the proportion of functional sequences inside the target, $P_{ubt}$, is computed using $P_{tol}$ (section A in S1 Text):


$$P_{ubt}={\left(P_{tol}\right)}^{n_{b}}.$$ (9)

The upper bound to the proportion of functional sequences in all of sequence space, $P_{ub}$, is the composite probability of a randomly selected sequence residing inside the target times the probability of a randomly selected sequence inside the target being functional:


$$P_{ub}=P_{t}\cdot P_{upt}.$$ (10)

### Rarity calculations for proteins using *P*_*tol*_ and *ΔΔG* data

If P(n) is not known or does not display negative epistasis, a target cannot be defined. Instead, the method prescribed uses $P_{tol}$ to directly calculate $P_{ub}$ by replacing $n_{b}$ in Eq. 9 with L:


$$P_{ub}={\left(P_{tol}\right)}^{L}.$$ (11)

The most important data for this method are also experimental measures of $P_{tol}$ in proteins that have accumulated sufficient mutations to move beyond the initial stability threshold. This equation will always yield a larger value than Eq. 10, as it assumes that *P*_*fs*_ outside the target is the same as inside, when the former is effectively zero.

If neither P(n) nor $P_{tol}$ are known, the method prescribed uses the percentage of non-destabilizing mutations, $P_{nd}$, as a surrogate for $P_{tol}$ in Eq. 11:


$$P_{ub}={\left(P_{nd}\right)}^{L}.$$ (12)

This substitution is justifiable due to the close connection between destabilizing and deleterious mutations [22–24]. For proteins mutated beyond their stability limit, any additional mutation with ΔΔG above a threshold, $G_{th}$, will generally destabilize the protein sufficiently to disable it; mutations with ΔΔG values below this cutoff are generally tolerated. The correlation is not perfect but sufficiently large to yield a reliable upper bound to $P_{fs}$. The essential data for this method are ΔΔG data that allow for the generation of an accurate probability distribution. Additional important data include details about the protein’s function, such as its mechanistic complexity, which allow for an accurate estimate of $G_{th}$.

The stability cutoff can also be estimated using β-lactamase as a benchmark since its cutoff can be derived from the available ΔΔG data for the protein. The greater a protein’s required specificity to perform its function, the lower $G_{th}$, since drops in stability more quickly disable proteins that operate within tighter constraints. If a protein’s functional specificity is at least as great as that for β-lactamase, β-lactamase’s $G_{th}$ can be used in rarity calculations. The value of $P_{nd}$ can then be determined, if sufficient ΔΔG data is available, by calculating the percentage of mutations with ΔΔG values below $G_{th}$. The rarity directly follows from Eq. 12 (section C in S1 Text).

## Results

### Application to epistatic proteins

The three calculation steps described above for the epistatic protein method were performed on β-lactamase, GFP, and HisA. As noted, β-lactamase serves as both a model for implementing the method and a calibration standard for evaluating other proteins. In contrast, GFP and HisA perform substantially more complex functions, which is reflected in their higher estimated β values.

#### Application to β-lactamase.

**Fig 3:**
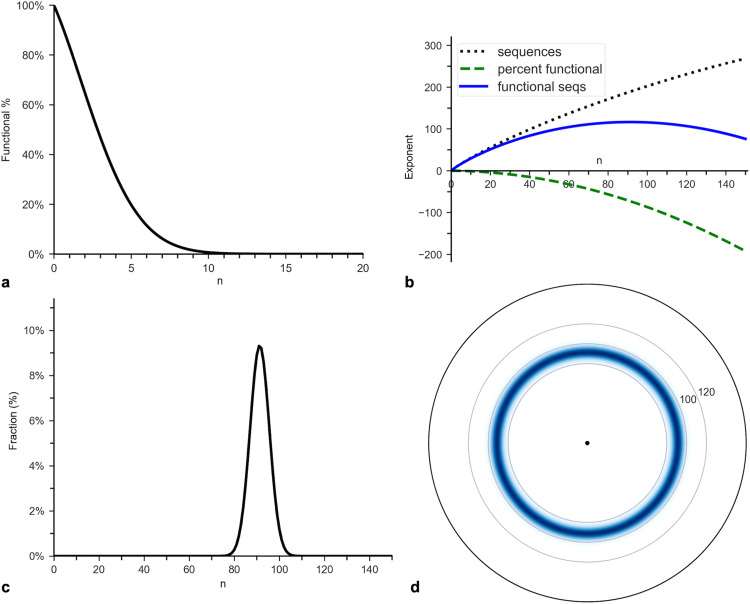
Distributions of functional sequences for β-lactamase. **(a)** Probability function, P(n), for β-lactamase in *E. coli* reported by Bershtein et al. (2006). It represents the percentage of clones with n nonsynonymous mutations that could survive in a 12.5 mg/ml solution of ampicillin. The investigators reported that the data best fit the hyper-exponential model with α =.104 and β = 0.019: $P(n=\;exp(\text{0}.\text{1}\text{0}\text{4}n\;-\;\text{0}.\text{0}\text{1}\text{9}n^{\text{2}})$. The x-axis is rescaled from the original to represent only nonsynonymous mutations, which are approximately 69% of all mutations. **(b)** Log-linear plot of the number of sequences (dotted line), P(n) (dashed line), and the number of functional sequences (solid line) that are n amino acid alterations away from the wildtype. The y-axis is the log of the functions’ values. **(c)** Plot of the probability distribution function, PDF(n), for functional sequences (Eq. 2). **(d)** Functional sequence heat (FSH) map for β-lactamase derived from PDF(n).

To determine the rarity of β-lactamase, the parametersg for the calculations were derived from the data reported by Bershtein et al. (2006) on TEM-1 β-lactamase from *E. coli* (L = 263) [35]. The first calculation step used the P(n) reported by the investigators for trials that applied the lowest cutoff for protein function. The reported P(n best fit the hyper-exponential function (Eq. 1) with α = 0.104 and β = 0.019 (Fig 3a). I ran the data listed in Table 1 in their Supplementary Information through the Python function curve_fit to identify the error bars on α and β, yielding $\sigma_{\alpha }$ = 0.02 and $\sigma_{\beta }$ = 0.004. The function also generated the coefficient of determination ($R^{\text{2}}$)—the percentage of variance explained—for both the hyper-exponential model and the exponential model for comparison. $R^{\text{2}}$ = 98.1% for the hyper-exponential and 96.0% for the exponential, demonstrating the superiority of the former.

**Table 1:** Variables used in the study are listed in order of appearance in the main text.

Variable	Meaning
$P_{fs}$	Proportion of functional sequences
n	Number of altered amino acids
${\Delta G}_{U-N}$	Stability of protein
ΔΔG	Change in stability from a mutation
$n_{b}$	Maximum n in target
P(n)	Probability of protein functioning with n alterations
$P_{tol}$	Percentage of tolerated mutations
*α*	Initial exponential decay
*β*	Rate of change of exponential decay (epistasis)
$P_{th}$	Probability threshold for a successful search
$T_{m}$	Maximum number of trials
c	Target size in nucleotide sequence space
$c_{a}$	*c* value in amino acid sequence space
PDF(n)	Probability distribution for functional sequences
$N_{seq}\left(n\right)$	Number of sequences with n alterations
$N_{FS}$	Total number of functional sequences
L	Protein Length
$n_{0}$	Number of initial mutations
$P_{t}$	Probability of sequence residing in target
d	Average discovery time of target
$P_{dt}$	Probability of discovering target
$P_{ubt}$	Upper bound to $P_{fs}$ in target
$P_{ub}$	Upper bound to $P_{fs}$
$P_{nd}$	Percentage of non-destabilizing mutations
$G_{th}$	Threshold for destabilizing mutations
A	Coefficient in exponential equation
B	Base in exponential equation
$\sigma_{\alpha }$, $\sigma_{\beta }$	Standard deviations of α and β
$d_{m}$	Average discovery time for one of m targets
$c_{min}$	Minimum size target that could be discovered

Since PDF(n) is constructed by multiplying P(n) by $N_{seq}(n)$, it peaks for n much larger than 0. The rate at which $N_{seq}(n)$ increases, as n increases, steadily declines. In contrast, the rate at which P(n) decreases steadily rises (Fig 3b). The opposing trends cause their product to peak at a value much larger than 0 but much smaller than the c = 1/3 boundary.

PDF(n) places most functional sequences between n = 80 and n = 105 (Fig 3c; see Table S1 in S1 File). The outer boundary corresponds to $c_{a}$ = 40%, which is equivalent to c = 21% (see Table S2 in S1 File). As mentioned, the distribution is not quantitatively but qualitatively accurate, so it is only used in qualitative comparisons with other proteins. Nevertheless, the boundary derived from P(n illustrates how choosing a c = 1/3 target, in all likelihood, overestimates the actual boundary.

**Fig 4:**
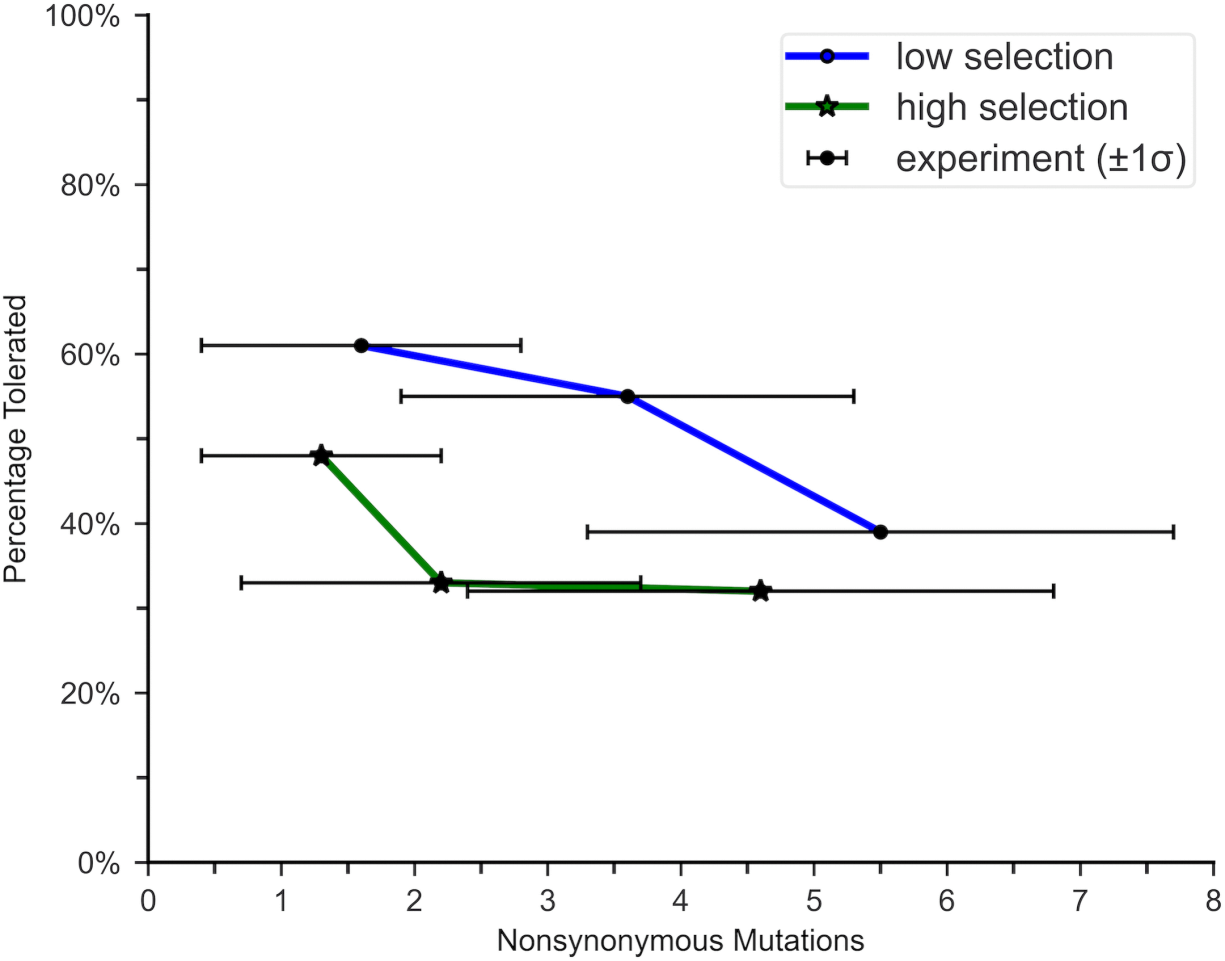
Percentage of tolerated mutations for β-lactamase. The graph displays the percentage of tolerated nonsynonymous mutations in β-lactamase after 2, 5, and 10 rounds of mutagenesis, as reported by Bershtein et al (2006). Selection was applied after each round. The results are presented for trials using 12.5 mg/ml of ampicillin (low selection) and 250 mg/ml of ampicillin (high selection). The horizontal bars represent the spread in the number of amino acid changes after the given round. Under high selection, $P_{tol}$ quickly dropped to approximately 1/3 and then plateaued. Under low selection, $P_{tol}$ was quickly approaching 1/3, but rounds of mutagenesis were ceased before the value plateaued.

The second calculation step used measurements of $P_{tol}$ obtained from trials that applied selection between rounds. The reported $P_{tol}$ quickly plateaued or approached 1/3 for all trials (Fig 4). For those with the lowest selection pressure (i.e., the lowest concentration of antibiotic), $P_{tol}$ would likely have dropped to 1/3 around $n_{\text{0}}$ = 7, if an additional round of mutagenesis were performed. Based on these results, $n_{b}$ was calculated using Eq. 3 for $P_{tol}$ = 1/3, $n_{\text{0}}$ = 7, and P(7 = 0.19, yielding $n_{b}$ = 89.

For a more conservative estimate, the second calculation step was repeated using P(n). Since the function was determined using data for clones with up to 14 nonsynonymous (amino acid altering) mutations, $n_{b}$ was calculated using $n_{\text{0}}$ = 14, P(14) = 0.006, and $P_{tol}$ estimated as P(15)/P(14)≈1/2. These values yield $n_{b}$ = 139. Both estimates fall within the c = 1/3 target, corresponding to $n_{b}$ = 150 aa (0.57·263). The FSH map (Fig 3d) is consistent with this choice of target.

Calculations 2a and 2b used c = 1/3 to yield $P_{t}$ = 10^-74^ (Eq. 4) and d = 10^133^ (Eq. 6). The latter result leads to $P_{dt}$ = 10^-95^ (Eq. 8). Repeating 2b with the very conservative c = 1/2 yields d = 10^51^ (Eq. 7), resulting in $P_{dt}$ = 10^-13^.

Calculation step 3 used c = 1/3 and $P_{tol}$ = 1/3 to yield $P_{ubt}$ = 10^-72^ (Eq. 9). The composite rarity estimate is $P_{ub}$ = 10^-146^ (Eq. 10). Repeating the calculation with the very conservative $P_{tol}$ = 1/2 yields $P_{ubt}$ = 10^-45^ and $P_{ub}$ = 10^-119^.

#### Application to GFP.

**Fig 5:**
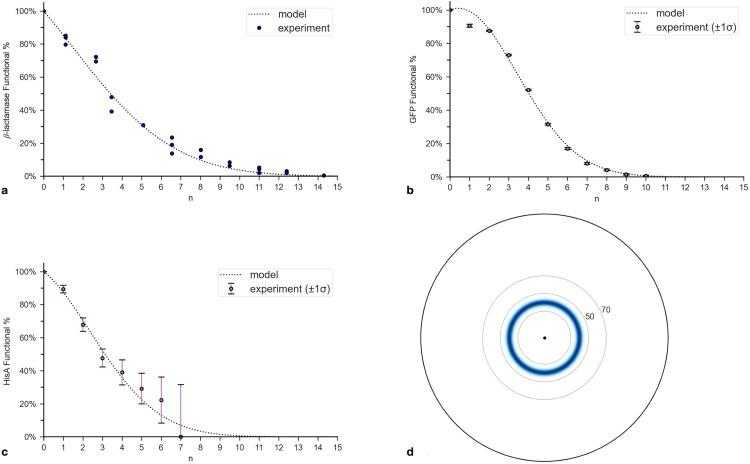
Distributions of functional sequences for β-lactamase, GFP, and HisA. All protein data were fit to the hyper-exponential model (Eq. 1). **(a)** P(n) for β-lactamase, which was generated by curve-fitting the experimental data from Bershtein et al. (2006). **(b)** P(n) for green fluorescent protein (GFP) from *Aequorea victoria*, which was generated by curve-fitting the experimental data from Sarkisyan et al. (2016). **(c)** P(n) for HisA, which was generated by curve-fitting the experimental data from Lundin et al. (2018). **(d)** FSH map for GFP.

To estimate the rarity of GFP, the parameters for the calculations were determined using the data reported by Sarkisyan et al. (2016) on GFP from *Aequorea victoria* (*L* = 238). For the first calculation step, P(n) was derived using the data on the protein’s fluorescence in clones as a function of nonsynonymous mutations [26]. The data sets are available at Figshare: http://dx.doi.org/10.6084/m9.figshare.3102154. The percentages of functional proteins up to n = 10 were fit to Eq. 1 using curve_fit, yielding α = −0.047 ± .003 and β = 0.054 ± .001. The data fit the model even better than for β-lactamase, except for n = 1 (Fig 5a and 5b). $R^{\text{2}}$ for the hyper-exponential is 99.4%, as compared to 86.9% for the exponential, confirming the hyper-exponential’s validity. The model predicts that most functional sequences reside between n = 30 and n = 50 (see Table S3 in S1 File).

For the second calculation step, the upper bound to the target size, $n_{b}$, was determined using P(n for the largest reported n, which is 10 mutations. The values input into Eq. 3 were $n_{\text{0}}$ = 10, P(10) =.007, $P_{tol}$ = P(11)/P(10)≈1/3  yielding $n_{b}$ = 89. Using the very conservative $P_{tol}$ = 1/2 yields $n_{b}$ = 136. Both estimates fall within or closely match the c = 1/3 boundary, corresponding to $n_{b}$ = 135 aa (0.57·238). The FSH map (Fig 5d) is consistent with this choice of target.

Calculations 2a and 2b used c = 1/3 to yield $P_{t}$ = 10^−68^ (Eq. 4) and d = 10^122^ (Eq. 6). The latter result leads to $P_{dt}$ = 10^−84^ (Eq. 8). Repeating 2b with the very conservative c = 1/2 yields d = 10^47^ (Eq. 7), resulting in $P_{dt}$ = 10^−9^.

Calculation step 3 used c = 1/3 and $P_{tol}$ = 1/3 to yield $P_{ubt}$ = 10^-64^ (Eq. 9). The composite rarity estimate is $P_{ub}$ = 10^-132^ (Eq. 10). Repeating the calculation with the very conservative $P_{tol}$ = 1/2 yields $P_{ubt}$ = 10^-41^ and $P_{ub}$ = 10^-109^.

#### Application to HisA.

To assess HisA’s rarity, the parameters for the calculations were determined using the results from studies on HisA from *E. coli* (L = 254) and using β-lactamase as a standard. For the first calculation step, P(n) was derived from the Lundin et al. (2018) data in Table 6A (Supplementary Information) on HisA’s activity in clones as a function of nonsynonymous mutations [36]. The table provided the percentage of functional sequences up to n = 8. Only one clone had 8 mutations, so that data point was not included. The percentages, copied into Table S4 within S2 Table (Supplementary Information), were fit to Eq. 1 using curve_fit, yielding α = 0.096 + /- 0.033 and β  = 0.039 + /- 0.013 (Fig 5c). $R^{\text{2}}$ for the hyper-exponential is 96.9%, as compared to 93.9% for the exponential, again confirming the hyper-exponential’s validity. The model predicts that most sequences reside between n = 40 and n = 60 (see Table S4 in S1 File).

The second calculation step used β-lactamase’s $P_{tol}$ = 1/3, as HisA performs a more complex function and exhibits a larger estimated β (section C in S1 Text). By extension, $n_{\text{0}}$ was set to where P(n+1)/P(n) first approximates 1/3, which is n = 12. The values input into Eq. 3 were $n_{\text{0}}$ = 12, P(12= 0.0011, $P_{tol}$ = 1/3 yielding $n_{b}$ = 90. A more conservative estimate used P(n) within the bounds of the experimental data. The values input into Eq. 3 were $n_{\text{0}}$ = 7, P(7) = 0.076, $P_{tol}\;=\;P(\text{8})/P(\text{7})\approx \text{1}/\text{2}$, yielding $n_{b}$ = 136. Both estimates fall within the c = 1/3 target, corresponding to $n_{b}$ = 144 aa (0.57·254).

Calculations 2a and 2b used c = 1/3 to yield $P_{t}$ = 10^−72^ (Eq. 4) and d = 10^130^ (Eq. 6). The latter result leads to $P_{dt}$ = 10^−92^ (Eq. 8). Repeating 2b with the very conservative c = 1/2 yields d = 10^48^ (Eq. 7), resulting in $P_{dt}$ = 10^−10^.

Calculation step 3 used c = 1/3 and $P_{tol}$ = 1/3 to yield $P_{ubt}$ = 10^-69^ (Eq. 9). The composite rarity estimate is $P_{ub}$ = 10^-141^ (Eq. 10). Repeating the calculation with the very conservative $P_{tol}$ = 1/2 yields $P_{ubt}$ = 10^-44^ and $P_{ub}$ = 10^-116^.

### Application to proteins with reported *ΔΔG* distributions

**Fig 6:**
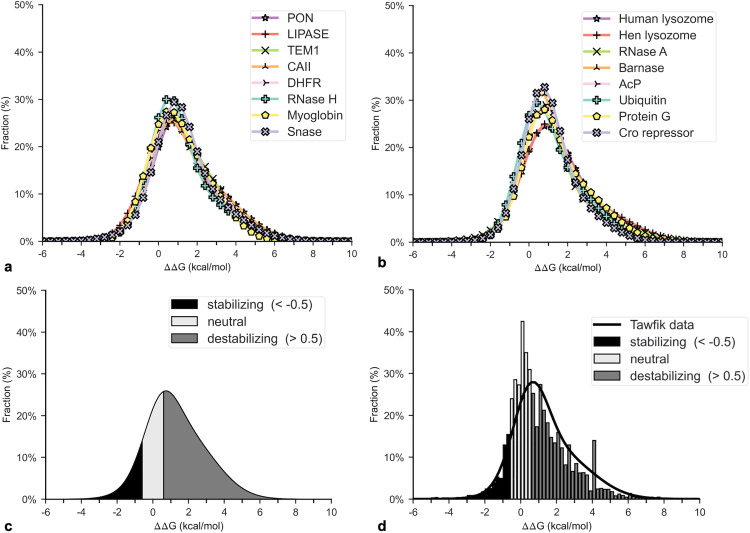
Probability distributions of *ΔΔG.* **(a and b)** Probability distributions Tokuriki and Tawfik (2007) reported for 16 natural proteins. **(c)** Probability distribution for β-lactamase as reported by Tokuriki and Tawfik (2007). The regions are identified where mutations are stabilizing (black), neutral (light grey), and destabilizing (dark grey) based on −1/2 kcal/mol and 1/2 kcal/mol cutoffs, which correspond to $P_{nd}$ = 1/3. **(d)** Distribution of all ΔΔG values recorded in FireProtDB. The mean distribution from Tokuriki et al. (2007) is superimposed for comparison. The mean distribution uses the averages of the means and standard deviations of the 16 natural proteins.

This study applied the method incorporating ΔΔG data to calculate the rarity for the 16 natural proteins analyzed by Tokuriki et al. (2007) using their reported ΔΔG probability distributions (Fig 6a and 6b), which closely fit bi-Gaussians [37]. The investigators listed the parameter values for each distribution, including β-lactamase. This study used the β-lactamase distribution to determine the *ΔΔG* cutoff between destabilizing and non-destabilizing mutations that coincides with $P_{tol}$ = 1/3 as reported by Bershtein et al. (2006). The distribution was integrated from negative infinity to where the integral equals $P_{tol}$, yielding $G_{th}$ close to 0.5 kcal/mol (Fig 6c). This value is a common choice for the destabilization cutoff in protein studies [38,39].

The 1/2 kcal/mol cutoff was used to calculate $P_{nd}$ for the other 15 proteins by integrating their distributions from negative infinity to the cutoff. The ΔΔG distributions are very similar, so the derived $P_{nd}$ values are also similar. The proteins’ $P_{nd}$ directly yielded their $P_{ub}$ (Eq. 12). The calculation was repeated with the more conservative 1 kcal/mol cutoff since it is also a common choice in protein studies [1,40].

For the lower cutoff, $P_{nd}$ is below $P_{th}$ for 13 of the 16 proteins. For the higher cutoff, *P*_*ub*_ is below $P_{th}$ for 10 proteins. The proteins that are not below $P_{th}$ have lengths below 125 aa. For the higher cutoff, $P_{ub}$ is below the multicellular eukaryotic threshold for all the proteins except Cro repressor (59 aa). The rarity estimates are listed in Table 2.

**Table 2:** *P*_*ub*_ for proteins in Tokuriki et al. (2007). The upper bound to the proportion of functional sequences, $P_{up}$, for each protein was calculated using Eq. 12. Values for $P_{nd}$ were derived from the reported ΔΔG distributions reported by Tokuriki et al. (2007) using both the 0.5 kcal/mol and 1.0 kcal/mol cutoffs between destabilizing and non-destabilizing mutations.

Protein	Length	*P_nd_* (0.5)	*P_ub_* (0.5)	*P*_*nd*_ (1.0)	*P*_*ub*_ (1.0)
Recombinant serum paraoxonase 1	332	31%	10^-171^	43%	10^-121^
Lipase	285	39%	10^-118^	51%	10^-83^
β-lactamase	263	34%	10^-124^	47%	10^-87^
Human carbonic anhydorase II	259	33%	10^-124^	46%	10^-88^
Dihydrofolate reductase	159	34%	10^-74^	48%	10^-51^
Robinuclease H	155	39%	10^-64^	53%	10^-42^
Myoglobin	151	38%	10^-64^	52%	10^-44^
*Staphylococcus* nuclease	136	32%	10^-68^	46%	10^-46^
Human lysozome	130	31%	10^-66^	43%	10^-47^
Hen lysozome	129	29%	10^-69^	42%	10^-49^
Ribonuclease A	124	37%	10^-54^	50%	10^-37^
Barnase	108	36%	10^-48^	52%	10^-31^
Acylphosphatase	98	33%	10^-48^	47%	10^-32^
Ubiquitin	76	40%	10^-31^	54%	10^-21^
Protein G	61	31%	10^-31^	45%	10^-21^
Cro repressor	59	37%	10^-26^	53%	10^-17^

### Application to proteins in FireProtDB

**Fig 7:**
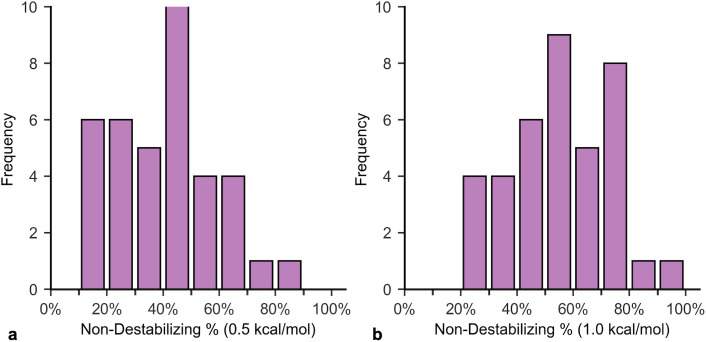
Histograms of *P*_*nd*_ for proteins in FireProtDB. The histograms include $P_{nd}$ for the 38 proteins with at least 30 recorded ΔΔG values. The histograms used stability cutoffs of **(a)** 0.5 kcal/mol and **(b)** 1.0 kcal/mol. Lower $P_{nd}$ corresponds to more extreme rarity for proteins with similar lengths.

This study applied the method incorporating ΔΔG data to calculate the rarity (Eq. 12) of the 38 proteins in FireProtDB that had at least 30 recorded ΔΔG values. $P_{nd}$ was estimated as the percentage of ΔΔG entries for each protein below the 0.5 kcal/mol cutoff. The average $P_{nd}$ with the 0.5 kcal/mol cutoff is 41%. For more conservative estimates, the calculation was repeated using the 1.0 kcal/mol cutoff, resulting in an average $P_{nd}$ of 55%. Histograms of the $P_{nd}$ values for both cutoffs are shown in Fig 7. The percentage of proteins with $P_{ub}$ above $P_{th}$ is 21% for the 0.5 kcal/mol cutoff and 47% for the 1.0 kcal/mol cutoff. For the multicellular eukaryotic threshold, the percentages drop to 3% (0.5 kcal/mol) and 21% (1.0 kcal/mol). All programs used in the analysis and data are available on GitHub: https://github.com/drbjmiller/Protein-Schema.

## Discussion

The results of applying PRISM to various proteins demonstrate its broad applicability. Additionally, previous research supports the general reliability of the different methods.

### Low-specificity functions

The method of generating libraries of peptides or polypeptides and directly measuring the percentage that perform the function of interest has proven effective in previous studies. Keefe and Szostak (2001) constructed random libraries of 80 aa polypeptides and identified those that bound to ATP, which was roughly 1 in 10^12^ sequences [10]. Knopp et al. (2019) constructed random libraries of 50 aa peptides and determined that 1 in 10^8^ sequences embedded into cell membranes and blocked the uptake of an antibiotic [41]. Both studies adhere to the PRISM guideline of using random libraries exclusively for low-specificity functions. Polypeptide sequences can readily bind to molecules, and membrane embedding simply depends on a sufficient proportion of hydrophobic side chains exposed on the protein’s surface.

Such activities contrast sharply with true enzymes that use specific sidechains to perform targeted manipulations on substrates stabilized by an active site with a specific geometry. No random library has ever generated sequences that could perform a function that requires such tight constraints. This limitation is consistent with PRISM’s decision tree that prescribes statistical methods for calculating the rarity of proteins that perform high-specificity functions.

### Proteins displaying negative epistasis

β-Lactamase, GFP, and HisA represent ideal test cases for the PRISM method for proteins displaying negative epistasis. They encompass a broad range of functional complexity and have sufficient experimental data available to identify $P_{\text{tol}\;}$ and construct P(n) more reliably than for most proteins. For each, the hyper-exponential model closely matches the experimental data as indicated by its high coefficient of determination ($R^{\text{2}}$), small residuals for most data points, and better fit than the exponential model. The HisA data show the largest error bars, resulting in the largest $\sigma_{\beta }$. Despite this increased uncertainty, all model predictions remain within the error bounds of their respective data points, and β is three standard deviations above zero.

In addition, several studies on diverse proteins indicate that negative epistasis persists for large n [42,43], so the hyper-exponential model should also apply for large n, at least qualitatively. This result, together with the excellent fit of the three proteins’ P(n) to hyper-exponential functions, indicates that many proteins’ functional sequences occupy a finite target in sequence space, enabling PRISM to generate reliable estimates of $P_{ub}$.

For β-lactamase, GFP, and HisA, the estimated $n_{b}$ fall within the c = 1/3 target (greater than 40% SI), even under extremely conservative assumptions. The predominance of negative epistasis further supports this conclusion. An undirected search would almost certainly fail to encounter a functional sequence beyond the defined targets for the three proteins. These findings align with previous studies identifying 40% SI as the threshold below which two sequences are unlikely to share the same function. For example, Tian and Skolnick (2003) demonstrated that similarities in enzyme function start to diverge quickly below 60% SI, and even the general type of function diverges below 40% SI [44].

The approach of qualitatively comparing the rarities of different proteins based on their β parameters and FSH maps is affirmed by the correspondence between the sizes of the three proteins’ β estimates and the complexity of their functions (section C in S1 Text). GFP displays much greater negative epistasis than β-lactamase (i.e., has a much larger β), resulting in functional sequences concentrating closer to the wildtype sequence, thus smaller targets and greater rarity in relation to its length (Fig 5d). This result is congruent with the greater complexity of GFP’s function; β-lactamase breaks a single bond, while GFP builds its own light-producing center through several internal chemical steps [45]. As an important note, the calculated $P_{ub}$ for GFP is larger than for β-lactamase since negative epistasis was ignored for large n and GFP is shorter, which is why FSH maps are important in qualitative comparisons.

Likewise, HisA’s estimated β is significantly larger than for β-lactamase, with nonoverlapping error bars, indicating that HisA displays greater negative epistasis and thus more extreme rarity in relation to its length. Its sequences are predicted to reside closer to the wildtype. This conclusion is consistent with HisA exhibiting higher mechanistic complexity than β-lactamase, as it performs multiple intramolecular rearrangements rather than breaking a single chemical bond [46].

The accuracy of the method for calculating $P_{ubt}$ is strongly supported by the rarity estimate for the 153 aa domain of β-lactamase studied by Axe (2004). He reported that as low as 1 in 10^77^ sequences corresponds to a functional protein domain [47]. In comparison, Eq. 9 for $P_{ubt}$ with c = 1/3, $P_{tol}$ = 1/3, and 150 variable amino acids yields 1 in 10^72^ functional sequences. Axe sampled sequences close to the wildtype protein (n = 30), so he also measured rarity inside the target. The close correspondence of the exponents for roughly the same number of variable amino acids affirms the accuracy of the method.

The reliability of the method for calculating the composite $P_{ub}$ is supported by the Tian and Best (2017) reported rarities of 10 protein domains [2]. Their estimates are based on a residue-residue co-evolution and thermodynamic stability model. The composite $P_{ub}$ (Eqs. 3, 4, 9, 10) were calculated in this study for the 10 domains using the same parameters identified for β-lactamase: c = 1/3 and $P_{tol}$ = 1/3 (Table 3). As mentioned, β-lactamase performs a relatively simple function, so these parameters should often provide reliable upper estimates for $P_{fs}$. The Tian and Best (2017) $P_{fs}$ estimates were close to or more extreme than the $P_{ub}$ calculations for all domains except Titin I27 and TNfn3.

**Table 3:** Rarity calculations for proteins studied in Tian and Best (2017). $P_{fs}$ of the ten single-domain proteins studied by Tian and Best (2017) are listed. The lengths of the proteins were used to calculate the effective $P_{tol}$ values from Eq. 11 by substituting$P_{fs}$ for $P_{ub}$ and solving backwards. Each domain’s composite $P_{ub}$ was calculated for c = 1/3 and $P_{tol}$ = 1/3 (Eqs. 3, 4, 9, 10).

Protein	Length	*P_fs_*	*P* *_t_* *_ol_*	*P_ub_*
WW	35	10^-24^	21%	10^-21^
Villin	35	10^-33^	11%	10^-21^
NTL9	56	10^-54^	11%	10^-33^
IM7	87	10^-86^	10%	10^-49^
Titin I27	89	10^-38^	37%	10^-51^
TNfn3	90	10^-39^	37%	10^-51^
PDZ	94	10^-50^	29%	10^-53^
a-LA	123	10^-121^	10%	10^-69^
IFABP	131	10^-111^	14%	10^-74^
OmpA	171	10^-126^	18%	10^-95^

The two outliers perform lower-specificity functions [48,49], so PRISM would not have prescribed the method for high-specificity functions displaying prevalent epistasis. It would have instead prescribed the method using Eq. 11, which yields $P_{ub}$ values with exponents reasonably close to the reported values—approximately 10^-43^ versus the reported 10^-38^ and 10^-39^.

### Estimating *P*_*tol*_

PRISM methods rely heavily on accurate estimates of $P_{tol}$. Yet reported values for this crucial parameter vary widely. Some studies estimate that as many as 2/3 of mutations in some proteins do not disable function [4,50]. Such estimates are typically taken from proteins that have acquired relatively few amino acid changes, so they have not passed the protein’s stability threshold. Studies on proteins that have acquired numerous mutations often report results reflecting $P_{tol}$ values of 1/3 or lower.

Rockah-Shmuel et al. (2015) applied 17 rounds of mutagenesis with selection to M.HaeIII, a DNA methyltransferase [51]. They estimated that 67% of mutations were clearly deleterious and an additional 16% were likely deleterious, so $P_{tol}$ could be as low as 20%. Konaté et al. (2019) studied the extent to which orthologous protein sequences diverged on planetary timescales [51]. The investigators reported that the average *P*_*tol*_ for nearly all surveyed proteins was less than 25%, some as low as 10%. These studies indicate that the best choice of $P_{tol}$ for many proteins should be 1/3 or less, further supporting the choice of β-lactamase as a standard.

### Rarities derived from *ΔΔG* values

Tian and Best (2017) also affirm PRISM’s $P_{ub}$ calculations using ΔΔG distributions. The effective $P_{tol}$ was derived for each domain the investigators studied using their estimated rarity and solving Eq. 11 backwards. The effective $P_{tol}$ for the two outlier domains are comparable to those calculated from the Tokuriki et al. (2007) distributions for the 0.5 kcal/mol cutoff (Table 2), and the $P_{tol}$ for the other eight domains are significantly smaller. The effective $P_{tol}$ are also typically smaller than those calculated from the FireProtDB ΔΔG data. Here again, the methodology appears to generate conservative upper bounds as desired.

The validity of the *P*_*ub*_ calculations is further supported by Konaté et al. (2019). As described above, the investigators reported the average *P*_*tol*_ for most proteins as less than 25%. This value is smaller than the *P*_*nd*_ values derived from Tokuriki et al. (2007) and FireProtDB using the 0.5 kcal/mol cutoff, again affirming that calculated *P*_*ub*_ values represent conservative upper bounds.

To confirm the conclusions of rarity, each of the proteins from Tokuriki et al. (2007) and FireProtDB should be examined for its stability and specificity requirements. PRISM’s method employing ΔΔG data would likely yield inaccurate results for proteins with intrinsically disordered or low-complexity regions since they function under different stability requirements than globular proteins. The same holds for proteins that perform low-specificity functions as described below.

### Non-globular proteins and conformational variability

The current PRISM analysis primarily focuses on globular proteins, which generally follow relatively simple energy landscapes (Fig 1c). Yet landscapes can be more complex, containing additional local minima such as cis/trans proline isomers, disulfide isomers, or amyloid fibrils. Mutations, environmental conditions, and high protein concentrations can reshape these landscapes, either destabilizing the native basin or stabilizing alternative states [52].

When the free energy gap between the native and non-native conformations is reduced or reversed, alternative states may become more dominant, leading to functional loss. Within the PRISM framework, such shifts could alter the effective values of $P_{tol}$ and $G_{th}$, likely decreasing them, resulting in lower $P_{fs}$. Future expansions to PRISM could incorporate analyses of these alternative states to refine upper-bound estimates of rarity.

Accounting for alternative states is particularly important for metamorphic proteins, which can switch between fundamentally different structures through a small number of amino acid changes or simply a change in environmental conditions (Fig 1b, 1d). These proteins can be treated in two ways. If alternative folds perform biologically relevant functions independently, PRISM can be applied to each conformation to yield separate estimates of rarity. If a protein’s function depends upon fold switching occurring at precise times between highly specified structures, alternative folds must be analyzed collectively. For instance, KaiB, a core clock protein in cyanobacteria, undergoes a conformational switch from its ground-state to an alternative conformation that participates in the bacteria’s circadian cycle [53]. The protein’s function depends on the ground-state switching at the right rate to an alternative structure that can bind to another clock protein.

PRISM methods face added challenges when applied to intrinsically disordered proteins (IDPs) and proteins with intrinsically disordered regions (IDRs), since they do not adopt a single stable fold but instead fluctuate across a dynamic ensemble of conformations occupying shallow energy minima (Fig 1e). IDRs can be identified by such tools as IUPred3 (iupred3.elte.hu), which estimates residue-residue interaction energies to predict if a sequence can participate in a stable fold. These proteins and protein regions operate within very different stability constraints than globular proteins. They require greater conformational flexibility to perform functions such as directing precisely timed interactions, binding multiple partners, and supporting signal transduction [54].

In IDRs, deleterious and destabilizing mutations are not as tightly correlated. The former are less likely to further destabilize already unstable regions than to disrupt highly specified sequences, such as molecular recognition features (MoRFs) and short linear motifs (SLiMs). Deleterious mutations can still be common, as demonstrated by the fact that 21.7% of mutations that cause disease in humans are located within IDRs [55].

PRISM faces similar challenges when applied to proteins with low-complexity regions (LCRs), which also deviate from stable globular folds due to biased amino acid composition [56]. They often perform similar functions to IDRs, such as binding to multiple molecules and regulating signaling. They can be detected by statistical algorithms that measure sequence complexity or composition bias, such as the SEG algorithm [57].

The distinct features of IDRs and LCRs constrain the application of PRISM to them. The method that incorporates *ΔΔG* data is unlikely to yield reliable results. The extent to which these proteins display significant negative epistasis with accumulating mutations is uncertain; therefore, the method that relies on *P(n)* might also not be applicable. In contrast, the method that only uses *P*_*tol*_ could yield reliable results for both classes of proteins. For proteins with a mix of highly structured regions and IDRs or LCRs, *P*_*tol*_ could be calculated separately, and the *P*_*ub*_ for each section could then be multiplied together to estimate rarity.

### Generality of rarity

The results demonstrate that extreme rarity is common among proteins. $P_{ubt}$ for β-lactamase, GFP, and HisA fall well below $P_{th}$, and random searches could not plausibly discover even their c = 1/2 targets. The same applies to the $P_{ub}$ derived using proteins’ ΔΔG distributions. A significant percentage of them fall below $P_{th}$ even using the very conservative stability cutoff of 1.0 kcal/mol. The similarity in the ΔΔG probability distributions from Tokuriki et al. (2007) and the relatively low specificity requirements of β-lactamase suggest that $P_{fs}$ for most globular proteins of even modest length should fall below $P_{th}$.

Extreme rarity is likely very common among proteins longer than 200 aa. Solving Eq. 7 backwards demonstrates that the c = 1/2 target could not be discovered for proteins longer than 210 aa. A very large portion of proteins’ targets should reside well within the c = 1/2 target since it matches what is termed the twilight zone for sequence identity (20–25%). Proteins with lower sequence identity do not typically share the same general function or structure [34,58]. In addition, $P_{ubt}$ falls below $P_{th}$ for proteins above 200 aa for c = 1/2 and $P_{tol}$ as high as 1/2 (Eq. 9).

Extreme rarity should be almost universal for proteins larger than 300 aa that perform high-specificity functions. Solving Eq. 11 backwards for $P_{ub}$ = 10^-40^ and n = 300 yields $P_{tol}$ = 74%, which is higher than that reported for nearly any protein.

### Multiple targets for proteins with the same function

The discovery time could be reduced if multiple targets correspond to proteins that perform the same function (functional analogs); however, in practice, multiple targets would not significantly alter the results. Chatterjee et al. (2014) determined that the average discovery time is not substantially reduced if multiple targets reside in the search space [31]. They derived the following equation for the average search time, $d_{m}$, to discover one of m targets of size c corresponding to a protein of length L:


$$d_{m}=\frac{\text{1}}{m}\cdot e^{\text{6}L{\left(\text{3}/\text{4}-c\right)}^{\text{2}}}$$ (13)

It yields discovery times with exponents slightly lower than those generated by the equation for a single target, so comparative analyses should use the same equation. Eq. 13 has the advantage that the minimal discoverable target size can be directly calculated as a function of the maximum allowed search time. For a search limited to d trials, the smallest target, $c_{min}$, for a protein of length L that could realistically be discovered through an undirected search is the following:


$$c_{min}=\frac{\text{3}}{\text{4}}-\sqrt{\frac{\text{1}}{\text{6}L}\text{ln}\left(m\cdot d\right)}$$ (14)

The minimum target size does not change significantly with increasing m. For the set of parameters (L = 250, d = 10^40^, m = 1), $c_{min}$= 50%. If m increases to 10^10^, $c_{min}$ only decreases to 47%. As a more extreme example, the parameters (L = 1000, d = 10^40^, m = 1) yield $c_{min}$= 63%, while (L = 1000, d = 10^40^, m = 10^40^) yield $c_{min}$= 57%. Both changes are minor in the context of PRISM, given that the estimated target size is likely much larger than the actual target; for β-lactamase, c derived from its P(n) is approximately 21%, while the value used in estimates of rarity is 33%.

In addition, the number of possible targets is limited. Scaiewicz and Levitt (2018) reported that most proteins represent combinations of a finite number of *single domain architectures* (SDAs), which are related to protein domains. The number of identified SDAs is plateauing in the tens of thousands [59]. Consequently, the number of functional analogs should not be sufficiently large to change the order of magnitude of $d_{m}$ significantly.

This conclusion is reinforced by the fact that the number of functional analogs in bacteria and in multicellular eukaryotes is often on the order of 10 or less, such as independent serine protease families in bacteria and in vertebrates [60]. Yet, the number of bacteria that have existed on Earth is many orders of magnitude larger than complex animals, plants, and fungi [61]. If the discovery of functional analogs were assisted by sequence space being saturated with them, the number of analogs should be many orders of magnitude larger in bacteria than in complex organisms. The fact that the numbers are so similar suggests that a vast number of analog targets either do not exist or have no significant impact on their average discovery time.

### Future studies

PRISM can be used to qualitatively compare the rarity of diverse proteins. Experiments can measure P(n) for as large an n as feasible. Trials that do not apply selection between rounds of mutagenesis can generate both P(n) and ${\text{P}}_{\text{tol}}$, while experiments applying selection between rounds can also estimate ${\text{P}}_{\text{tol}}$. When P(n) is best described by a hyper-exponential model (Eq. 1), the β parameter provides an estimate of the degree of negative epistasis, and the resulting FSH map can be compared with that of other proteins.

If ${\text{P}}_{\text{tol}}$ is not known, ${\text{P}}_{\text{nd}}$ could be calculated using a protein’s ΔΔG data and the appropriate cutoff based on the protein’s functional specificity. The cutoff of 0.5 kcal/mol is a reasonable choice for proteins with specificity requirements at least as high as those for β-lactamase, and 1.0 kcal/mol should be reasonable for most proteins that perform non-trivial functions and do not contain extensive intrinsically disordered or low-complexity regions. The calculated ${\text{P}}_{\text{nd}}$ directly yields ${\text{P}}_{\text{ub}}$, which can be compared with values calculated for other proteins.

Information on rarity could identify which proteins in a pathogen might be most susceptible to attack, and it could predict which human proteins are most susceptible to mutations that degrade their function, resulting in disease. Rarity estimates can also help constrain theories on the origin of natural proteins. For instance, Miller (2024) explained how ${\text{P}}_{\text{fs}}$ can be used to identify the level of required biasing for continuous paths of functional sequences (CPFSs) to connect distinct proteins in sequence space [3].

Identifying such paths is essential for explaining the origin of many proteins, as the prevalence of extreme rarity often precludes hypotheses that rely on random searches due to excessive search times. Scenarios for the origins of modern proteins should instead identify ancestral proteins that reside within the same local region of sequence space [62] and identify interconnecting CPFSs. The origin of novel high-specificity proteins often requires natural selection to constrain the evolution of a protein to follow a CPFS, so it would only need to explore a minute fraction of sequence space.

For the same reasons, strategies for engineering novel proteins that perform high-specificity functions should start with proteins with similar structures that perform target functions at a low level or similar functions [63]. Modifying existing proteins to engineer target proteins can be aided by such tools as the sequence evolution with epistatic contributions (SEEC) model developed by Alvarez et al. (2021), which identifies evolutionarily relevant epistatic interactions between amino acids [64]. The model’s developers employed it to guide the modification of enzymes, thereby efficiently exploring the proteins’ fitness landscapes to enhance target functions.

PRISM is anticipated to become a valuable resource for advancing protein engineering and functional studies across multiple subdisciplines, including enzyme design, therapeutic protein development, and structural biology.

## Supporting information

S1 Text Supplementary information on calculations. (DOCX)

S1 File Data used to determine parameters in methods. (XLSX)
