# Supplementary material for: A methodology for calculating the rarity of diverse proteins based on functional specificity and thermodynamic stability
Source: PLoS One. 2025 Dec 29;20(12):e0339572. doi: 10.1371/journal.pone.0339572 (PMC12747414; doi:10.1371/journal.pone.0339572)
Supplement: S1 Text — (DOCX) [file pone.0339572.s001.docx]

**S1 Text**

**Supplementary Information on Calculations**

1. **Probability Calculations**

**Calculating PDF(n)**

The *P(n)* probability function (Eq. 1) directly yields the distribution of functional sequences as a function of *n* (Hamming distance from wildtype). Each alteration in a protein could replace the original amino acid by any one of the 19 others. The total number of sequences, *N_seq_(n)*, that are *n* alterations away from the wildtype sequence is therefore the number of combinations of *n* amino acids chosen from *L*, the protein’s length, multiplied by 19^n^:

$N_{seq}(n)=\left( \begin{matrix} L \\ n \end{matrix} \right){19}^{n}$ (S1)

The number of functional sequences, *N_fs_(n)*, for a given *n* is the number of sequences times *P(n)* (Eq. 1):

$N_{fs}(n)=\left( \begin{matrix} L \\ n \end{matrix} \right){19}^{n}e^{-\alpha-\beta n^{2}}$ (S2)

The probability distribution, *PDF(n)*, is *N_fs_(n)* divided by the total number of functional sequences, *N_FS_*:

$PDF(n)=\frac{N_{fs}(n)}{N_{FS}}$ (S3)

**Calculating Upper Bound to Rarity**

An upper bound to the proportion of functional sequences inside the target, *P_upt_*, or in all of sequence space if the target is unknown, *P_up_*, can be derived by assuming that *P_tol_* of the 20 standard amino acids at each of the variable positions corresponds to a functional protein. Inside a target, the number of variable positions equals *n_b_* since at least *L*–*n_b_* amino acids must match the center sequence. This assumption likely overestimates the probability since Cauchy’s mean theorem [1] states that the product of numbers with a given mean (*P_tol_* in this case) is less than or equal to the mean raised to the power of the number of values:

$p_{1}\cdot p_{2}\cdot\ldots{\cdot p}_{n}{\leq\left( P_{tol} \right)}^{n}$, (S4)

where *p_i_* is the percentage of amino acids at the *i^th^* variable position that corresponds to a functional protein. The value for *P_ubt_* or *P_ub_* equates to the upper bound to the product of the *p_i_* values, which yields Eq. 9 and Eq. 11. The actual percentage of tolerated mutations is likely smaller than the value shortly after the threshold due to negative epistasis, so *P_fs_* could be significantly smaller than this estimate.

1. **Protein Targets**

**Calculating Boundary**

The upper bound to the value of *n* for a target boundary, *n_b_*, is estimated using the percentage of tolerated mutations, *P_tol_*, after the first *n_0_* mutations drop the stability past the initial threshold. The calculation follows the same logic as in section A. The upper bound to the probability for a sequence being functional with *n* alterations is *P(n_0_)* times *P_tol_* raised to the *n*‑*n_0_* power:

$P\left( n \right)<$ $P\left( n_{0} \right)\cdot{(P_{tol})}^{n-n_{o}}$. (S5)

The value from *P(n_o_)* can be taken from either Eq. 1 or from the experimental data. The equation overestimates the probability for the reasons stated above. Solving for *n* after setting *P(n)* to *P_th_* yields Eq. 3.

The estimate of rarity in section A did not include *P(n_0_)* and took *P_tol_* to the power of *n* instead of *n*–*n_0_* since *n_0_* is usually much smaller than either *n_b_* or *L*. The extent that ignoring the stability threshold underestimates the probability is typically more than compensated by the extent to which ignoring negative epistasis overestimates it. This premise is strongly supported by Breen et al. (2012), who demonstrated that negative epistasis is pervasive as a wildtype protein sequence is continuously altered [2]. They estimated that as many as 90% of mutations that would initially be neutral or beneficial in a wildtype sequence would be detrimental in a significantly altered sequence. They based their conclusion on comparing orthologous proteins across diverse species. Even for proteins not demonstrating extensive epistasis, using Eq. Using S5 instead of Eq. 9 or Eq. 11 would not qualitatively change the results.

**Maximum Number of Trials**

Chatterjee et al. (2014) estimated the maximum number of independent searches possible by every organism on the planet by assuming an average global bacteria population of 10^30^, a mutation rate of 10^‑9^ per nucleotide, and an average gene length of 1000 nucleotides. These values correspond to 1 mutation occurring on average in a gene each generation in a population of one million bacteria. This value leads to a maximum number of independent searches per generation of *I* = 10^24^. They estimated the maximum number of generations by assuming a 20‑minute generation time over the history of the Earth, which leads to *T* = 10^14^. The maximum number of trials, *T_m_*, is then *IT*= 10^38^. The threshold probability, *P_th_*, below which a search could not likely find a target is a few orders of magnitude below the inverse of *T_m_*, yielding a *P_th_* on the order of 10^-40^. The *P_th_* for complex animals and plants is closer to 10^-20^ since they have much smaller populations and longer life cycles than bacteria [3].

**Applicability of Chatterjee et al. (2014)**

Chatterjee et al. (2014) directly relates to the origin of novel proteins. Many protein superfamilies reside in regions of sequence space that are completely disconnected from other regions containing other superfamilies [4,5]. Consequently, the first representatives of many superfamilies differ sufficiently in sequence and structure from their nearest relatives that the shortest path between the proteins’ common ancestor and at least one of them is equivalent to starting from a random sequence. The discovery time for the first representative of the superfamilies, and likely for many families, equates to starting from a random sequence and randomly altering nucleotides until the sequence enters the target region of another protein.

Chatterjee et al. (2014) also discovered that a starting sequence just a few steps away from the target would not find the target any faster on average than starting from a random sequence. This observation relates to another important discovery by the investigators. Genetic processes beyond single nucleotide changes, such as recombination, do not significantly lower the average discovery time. No matter where in sequence space an evolutionary event sends a mutating ORF, the average discovery time remains the same. Consequently, the results from Chatterjee et al. (2014) accurately represent the time required for evolutionary searches to discover many protein targets.

**Converting to Amino Acid Sequence Space**

Chatterjee et al. (2014) studied nucleotide sequence space, so I converted the *c* = 1/3 and 1/2 targets to the corresponding *c_a_* values in amino acid sequence space using a Python program. For some trials, the program randomly generated nucleotide sequences of length 300 and then randomly altered 1/3 or 1/2 of the nucleotides. For other trials, the starting sequence was the ORF of a TEM-1 β-lactamase. Some trials altered nucleotides with equal probability, while others used the nucleotide transition matrix determined experimentally for *E. coli* [6] both forward and backward in time. The reverse matrix was included to model moving toward the β‑lactamase target instead of mutating away from it. The resulting number of altered amino acids was then counted.

For each set of parameters, the program was run 100,000 times, and the average number of altered amino acids was recorded. Every trial yielded average *c_a_* values that were within 2% of the results of other trials. The lowest *c_a_* values were for starting with TEM-1 β-lactamase and using the reverse nucleotide transition matrix. Combining all runs, the c = 1/3 target in nucleotide sequence space corresponds in amino acid sequence space to a *c_a_* = 57 ± 3% target, and the c = 1/2 target corresponds to a *c_a_* = 77 ± 3% target.

**Deriving Exponential Functions for Discovery Time**

The discovery time equation for a 1/3 target was identified with an exponential regression calculation using the average discovery times calculated by Chatterjee et al. (2014), (*L*= 33.3, *d* = 1.28⋅10^65^) and (*L* = 333, *d* = 5.89⋅10^170^), and using the point (*L* = 1, *d* = 2). Note that *L* = *N*/3, where *N* is the number of nucleotides. The exact value for the last point does not affect the results. The data closely fit Eq. 5 for *A* = 2.21 and *B*= 3.25. The same procedure applied to the data for the *c* = 1/2 target yields *A* = 5.39 and *B*= 1.56.

1. **Calculating Rarity from** *ΔΔ****G* Distributions**

**β-lactamase as Benchmark**

β-lactamase represents a helpful benchmark in evaluating the appropriate choice of *ΔΔG* cutoff for calculating *P_nd_* since the enzyme performs a relatively simple function. Proteins that perform more complex operations or maintain greater causal control over molecular manipulations require a larger quantity of functional information in their sequences [7], and more information-rich sequences are less probable [8]. In turn, lower probability corresponds to smaller *ΔΔG* values disabling the protein and thus a lower cutoff.

β-lactamase performs the relatively simple task of breaking a single bond in an antibiotic molecule. The same function can be achieved by catalytic antibodies (abzymes) generated in a random library of only a few billion in size [9]. The more complex catalytic activity displayed by many enzymes cannot be duplicated by abzymes, which only have randomized *variable* domains that bind to antigens. The *constant* regions, which provide structural support for the *variable* regions, always remain the same. An abzyme could break apart an antibiotic molecule, as this process does not require the same level of functional support from amino acids distant from the binding site as other enzymes [10].

In contrast, enzymes like topoisomerases or polymerases operate within far tighter constraints [11,12]. Their functions depend on precise interactions between multiple domains, and they depend on multiple highly coordinated conformational changes. They also require the precise positioning and orientation of multiple amino acids. The numerous tight constraints correspond to far more extreme rarity.

Since the β-lactamase function is relatively simple, a natural question is why the rarity is still so extreme. The rarity is not entirely due to the catalytic function specific amino acids play in the active site, but it is also due to the rest of the enzyme maintaining the correct structure to position and orient those amino acids properly. For abzymes, the structural support for the binding site is provided by the *constant* regions. The proportion of sequences that could play that role is very small.

Most proteins require structural support, but they have additional constraints. In contrast to β‑lactamase, GFP performs a bioluminescent activity that depends on a higher level of coordinated support from multiple amino acids in different locations of the protein [13]. These constraints correlate with its higher β, entailing a lower cutoff for *P_nd_*. Similarly, HisA participates in an intermediate step in the synthesis of the amino acid histidine, where it performs a highly specific molecular transformation [14]. Namely, the enzyme detaches a hydrogen from one nitrogen molecule and attaches a hydrogen to another nitrogen. As expected, HisA has a larger β than β‑lactamase since it performs a more complex function, so it should also have a lower stability cutoff and be rarer.

Many enzymes and structural proteins also perform more complex tasks with higher specificity requirements than β-lactamase. For example, DNA methyltransferase transfers a methyl group from a methyl donor to cytosine in DNA, which represents a higher-specificity function than breaking a single bond in an antibiotic. Rockah-Shmuel et al. (2015) determined from 17 rounds of random mutagenesis applied to DNA methyltransferase in *Haemophilus aegyptius* (M.HaeIII) that possibly over 80% of mutations are deleterious [15]. Consequently, its *P_tol_* could be as low as 20%, again suggesting in comparison to β-lactamase a lower stability cutoff and more extreme rarity.

**FireProtDB Data**

I programmed a Python script to identify which proteins in FireProtDB had at least 30 unique *ΔΔG* entries to obtain statistically significant results. When multiple entries existed for the same amino acid transition (e.g., R🡪A at the 4^th^ aa), I replaced the entries with a single entry equaling the average of the different values. For each selected protein, I divided the number of entries with *ΔΔG* values below the cutoff by the total number of entries. Runs with higher and lower sample size cutoffs yielded very similar results.

I plotted the *ΔΔG* probability distribution for all the database entries (Fig 6d). The distribution looks very similar to those Tokuriki et al. (2007) reported for 16 globular proteins [16] (Fig 6a and 6b), so the data does not appear to be strongly biased toward higher or lower *ΔΔG*. The main difference is that the FireProtDB data has a higher percentage of neutral mutations, which might result from it including proteins with intrinsically disordered or low-complexity regions. Such regions have different stability requirements than globular proteins.

**References**

1. Cauchy A-L. Cours d’analyse de l’École Royale Polytechnique. Paris: Impr. royale Debure frères; 1821.

2. Bershtein S, Segal M, Bekerman R, Tokuriki N, Tawfik DS. Robustness–epistasis link shapes the fitness landscape of a randomly drifting protein. Nature. 2006;444: 929–932. doi:10.1038/nature05385

3. Breen MS, Kemena C, Vlasov PK, Notredame C, Kondrashov FA. Epistasis as the primary factor in molecular evolution. Nature 2012 490:7421. 2012;490: 535–538. doi:10.1038/nature11510

4. Bar-On YM, Phillips R, Milo R. The biomass distribution on Earth. Proc Natl Acad Sci U S A. 2018;115: 6506–6511. doi:10.1073/pnas.1711842115

5. Nepomnyachiy S, Ben-Tal N, Kolodny R. Global view of the protein universe. Proceedings of the National Academy of Sciences. 2014;111: 11691–11696. doi:10.1073/pnas.1403395111

6. Bray JE, Todd AE, Pearl FMG, Thornton JM, Orengo CA. The CATH Dictionary of Homologous Superfamilies (DHS): a consensus approach for identifying distant structural homologues. Protein Engineering, Design and Selection. 2000;13: 153–165. doi:10.1093/PROTEIN/13.3.153

7. Lee H, Popodi E, Tang H, Foster PL. Rate and molecular spectrum of spontaneous mutations in the bacterium Escherichia coli as determined by whole-genome sequencing. Proceedings of the National Academy of Sciences. 2012;109: E2774–E2783. doi:10.1073/PNAS.1210309109

8. Griffiths PE, Pocheville A, Calcott B, Stotz K, Kim H, Knight R. Measuring Causal Specificity. https://doi.org/101086/682914. 2015;82: 529–555. doi:10.1086/682914

9. Hazen RM, Griffin PL, Carothers JM, Szostak JW. Functional information and the emergence of biocomplexity. Proceedings of the National Academy of Sciences. 2007;104: 8574–8581. doi:10.1073/pnas.0701744104

10. Shahsavarian MA, Chaaya N, Costa N, Boquet D, Atkinson A, Offmann B, et al. Multitarget selection of catalytic antibodies with β-lactamase activity using phage display. FEBS J. 2017;284: 634–653. doi:10.1111/febs.14012

11. Ricoux R, Mahy J-P. A New Generation of Artificial Enzymes: Catalytic Antibodies or ‘Abzymes.’ Comprehensive Natural Products II. 2010; 323–352. doi:10.1016/B978-008045382-8.00693-6

12. McKie SJ, Neuman KC, Maxwell A. DNA topoisomerases: Advances in understanding of cellular roles and multi-protein complexes via structure-function analysis. BioEssays. 2021;43: 2000286. doi:10.1002/BIES.202000286

13. Schier AC, Taatjes DJ. Structure and mechanism of the RNA polymerase II transcription machinery. Genes Dev. 2020;34: 465–488. doi:10.1101/GAD.335679.119

14. Barondeau DP, Putnam CD, Kassmann CJ, Tainer JA, Getzoff ED. Mechanism and energetics of green fluorescent protein chromophore synthesis revealed by trapped intermediate structures. Proc Natl Acad Sci U S A. 2003;100: 12111. doi:10.1073/PNAS.2133463100

15. Winkler ME, Ramos-Montañez S. Biosynthesis of Histidine. EcoSal Plus. 2009;3. doi:10.1128/ECOSALPLUS.3.6.1.9/ASSET/09EE4A4B-E315-4DA1-ACF1-F5BD1AB95CC7/ASSETS/GRAPHIC/3.6.1.9_FIG_004.GIF

16. Rockah-Shmuel L, Tóth-Petróczy Á, Tawfik DS. Systematic Mapping of Protein Mutational Space by Prolonged Drift Reveals the Deleterious Effects of Seemingly Neutral Mutations. PLoS Comput Biol. 2015;11: e1004421. doi:10.1371/JOURNAL.PCBI.1004421

17. Tokuriki N, Stricher F, Schymkowitz J, Serrano L, Tawfik DS. The Stability Effects of Protein Mutations Appear to be Universally Distributed. J Mol Biol. 2007;369: 1318–1332. doi:10.1016/j.jmb.2007.03.069
